# Laparoscopic resection of giant adrenal myelolipoma: A case report with review of literature

**DOI:** 10.1016/j.radcr.2024.07.072

**Published:** 2024-08-21

**Authors:** Rawa Bapir, Ismaeel Aghaways, Hadeel A. Yasseen, Rezheen J. Rashid, Shaho F. Ahmed, Ayman M. Mustafa, Nali H. Hama, Hawar A. Sofi Mohammed, Sanaa O. Karim, Fahmi H. kakamad, Berun A. Abdalla

**Affiliations:** a Smart Health Tower, Madam Mitterrand Street, Sulaymaniyah, Kurdistan, Iraq; b Sulaimani Teaching Hospital, Zanko Street, Sulaymaniyah, Kurdistan, Iraq; c Kscien Organization, Hamdi Street, Azadi Mall, Sulaymaniyah, Kurdistan, Iraq; d College of Medicine, University of Sulaimani, Madam Mitterrand Street, Sulaymaniyah, Kurdistan, Iraq; e Department of Radiology, Hiwa Hospital, Shorsh Street, Sulaymaniyah, Kurdistan, Iraq; f College of Nursing, University of Sulaimani, Madam Mitterrand Street, Sulaymaniyah, Kurdistan, Iraq

**Keywords:** Adrenal Gland, Myelolipoma, Tumor, Laparoscopy, Minimally invasive technique

## Abstract

Giant adrenal myelolipoma (AML) may cause severe symptoms. In contrast to the previous reports, laparoscopy may play a pivotal role in the management of giant AML. This report aims to discuss a case of giant AML managed successfully by laparoscopy. A 63-year-old male was found to have a giant (12 × 10 × 8 cm) left AML during a workup for left lower chest pain on imaging. laparoscopic excision of a left adrenal gland with the lesion was performed under general anesthesia. The patient was discharged from the hospital after 3 days uneventfully. AML is a benign tumor that is characterized by the presence of adipose tissue and hematopoietic elements. Myelolipomas are typically asymptomatic. AML diagnosis is based on imaging and blood workup. Small asymptomatic AML is usually managed conservatively, while symptomatic AML is managed with surgery. Even though an open approach is the standard option, laparoscopy, as a minimally invasive technique, in some centers may replace laparotomy. Laparoscopy can be a successful method for managing AML, even when they are large in size.

## Background

Adrenal myelolipoma (AML) is an uncommon noncancerous tumor of the adrenal gland. It is composed of mature adipose tissue and hematopoietic components, that looks like bone marrow [1]. It was only discovered at autopsies, equally between both genders, mainly in ages 50 to 60 of life, with a frequency of 0.08% to 2% [2]. However, with the advancements in radiological techniques like ultrasound, computed tomography (CT) scan, and magnetic resonance imaging (MRI), the incidental detection of myelolipoma has become more common, accounting for around 10%-15% of adrenal masses that are detected incidentally [3].

Most AMLs are typically small, with a diameter of 4 cm or less. Around 70% of these tumors are asymptomatic and are often detected unilaterally in the adrenal gland [4]. However, when the AML exceeds 10 cm in diameter, it is classified as a giant myelolipoma. Additionally, adrenal myelolipomas (AMLs) may be present alongside other adrenal disorders such as adrenal cancer, congenital deficiency of 21-hydroxylase enzyme, hypercortisolism, and adrenal medullary tumor, it is reported that approximately 5.7% of myelolipoma cases are associated with coexisting adrenocortical tumors, while congenital adrenal hyperplasia is seen in 10% of cases [5,6]. Adrenal myelolipoma is associated with a variety of health conditions, including, hyperlipidemia, obesity, high blood pressure, diabetes mellitus, and Cushing's disease [7]. It is reported that up to 19% of AMLs show histopathologic hemorrhagic changes. Spontaneous tumor rupture is observed in 4.5% of cases, and it is more common in larger tumors measuring 10-12 cm [6].

In most cases, small AMLs that are asymptomatic and less than 4 cm in size are managed conservatively. However, if symptoms are present, adrenalectomy is usually recommended [7,8]. There has been a rising trend of using minimally invasive techniques to manage giant AMLs with safe outcomes, although open adrenalectomy is still considered the most preferred method for managing giant AMLs [8].

This report aims to discuss a case of giant AML managed successfully by laparoscopy with a brief literature review.

## Case presentation

### Patient information

A 63-year-old male patient presented to Smart Health Tower (Sulaimani, Iraq) with the incidental finding of a left adrenal mass during a workup for left lower chest pain. Past medical history was significant for hypertension. The patient was taking Telmisartan, Amlodipine, Carvedilol, Crestor, and Clopidogrel for his underlying medical conditions. The patient had no previous surgical history.

### Clinical findings

Physical examination was unremarkable. Vital signs were normal. Body mass index was 31.1 kg/m^2^.

### Diagnostic approach

**Fig. 1:**
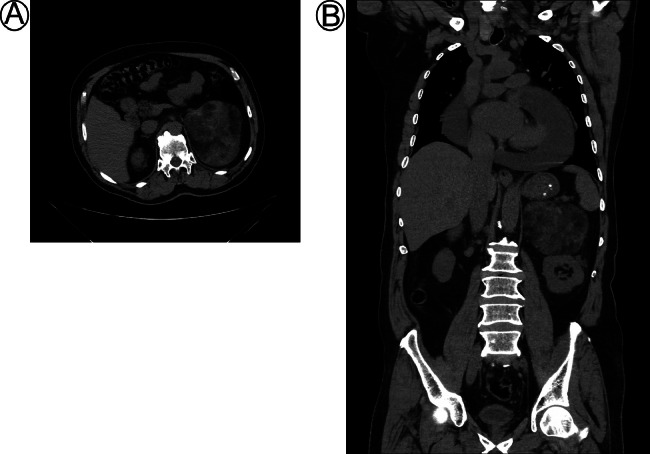
Abdominal Non contrast CT scan (A) axial section (B) coronal section revealed a well-defined lesion consisting of fat at left adrenal gland, the dimensions of the lesion were measured to be 12 × 10 × 8 cm in transverse x anteroposterior x craniocaudal dimensions, respectively.

A CT scan of the abdomen showed evidence of a 12 × 10 × 8 cm heterogenous density, fat-containing left retroperitoneal mass located above the left kidney. The mass occupies most of the left adrenal gland (Fig. 1). The plasma normetanephrine level was 146.5 pg/mL [normal range <196 pg/mL], the plasma metanephrine level was 43.9 pg/mL [normal range<65 pg/mL], and the dexamethasone suppression test was 44.05 nmol/L [normal range <50]. Biochemical tests including electrolytes and ionizing calcium, were all normal. Blood urea was 29 mg/dL (16–45), and serum creatinine was 0.79 mg/dL (0.7–1.2).

Echocardiography showed moderate to severe pericardial effusion mainly on the left ventricle (29 mm) and less than 10 mm on the right ventricle and apical areas, with a good ejection fraction (67%).

### Therapeutic intervention

**Fig. 2:**
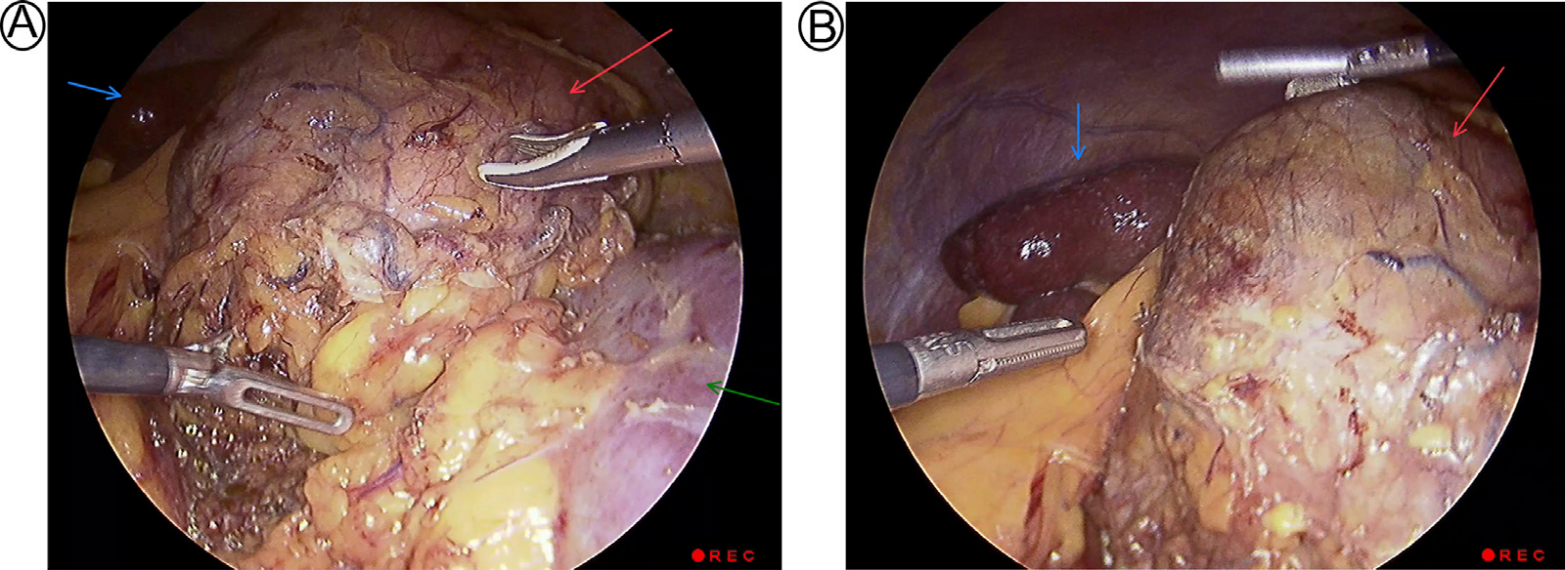
The laparoscopic view during surgery shows a mass (Red arrow) located adjacent to the left kidney (Green arrow) and spleen (Blue arrow).

**Fig. 3:**
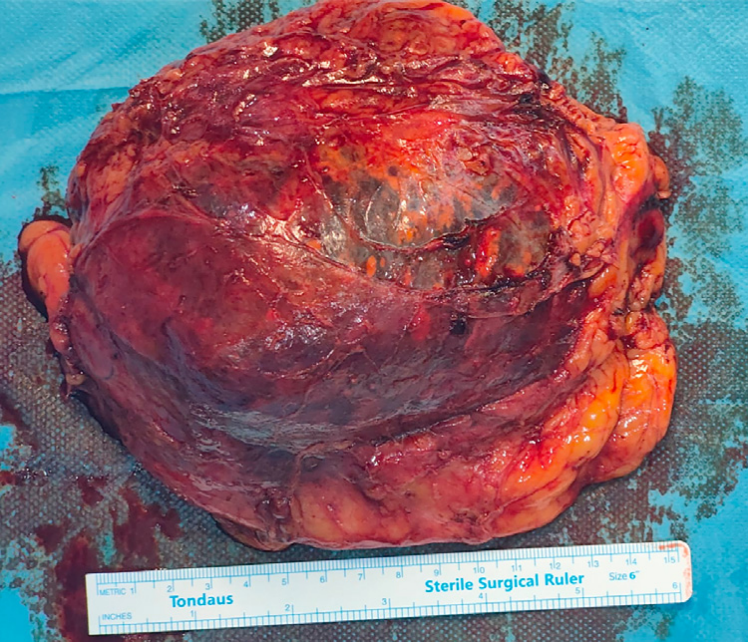
Gross appearance of the giant adrenal gland tumor.

Based on the size of the mass, which was greater than 10 cm, surgical removal of the left adrenal gland was necessary. Due to the presence of the adrenal mass, a pericardiocentesis procedure to drain the pericardial effusion was attempted but was unsuccessful as it caused compression and blocked access to the pericardial effusion. As a result, the patient was put on treatment for 2 weeks to stabilize his cardiac condition and optimize his overall health before proceeding with the surgical intervention. Later, the left adrenal gland mass was surgically removed through a laparoscopic left adrenalectomy procedure (Fig. 2). The laparoscopic method utilized 4 ports for visualization and excision of the adrenal mass, and the surgical team retrieved the specimen via a Pfannenstiel incision using endo-pouch, then the mass was sent for histological examination (Fig. 3).

**Fig. 4:**
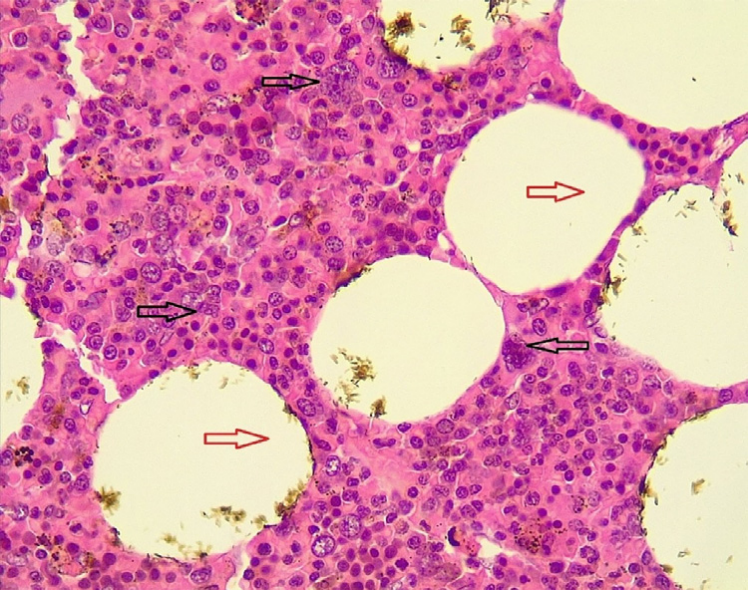
Mixture of mature adipocytes (red arrows) and extramedullary trilineage hematopoietic cells with a markedly increased number of megakaryocytes (black arrows).

The patient was discharged from the hospital after 3 days following an uneventful postoperative course. Histopathological examination confirmed that the adrenal mass was adrenal myelolipoma (Fig. 4).

### Follow-up and outcome

After 2 months of follow-up, no significant complication was observed.

## Discussion

Myelolipoma is a rare and benign tumor of the adrenal gland that was first characterized and described in 1905 by Gierkein and named in 1929 by Oberling [9]. It was first identified as a postmortem discovery. The prevalence was estimated to be between 0.08% and 0.4% in autopsies conducted in 1973. The incidence of AML has been steadily increasing over time due to the widespread use of noninvasive, high-resolution imaging diagnosis [10].

Adrenal myelolipoma is a condition whose exact cause is not yet known. However, several risk factors have been identified that contribute to its development, including degeneration, inflammation, stress, obesity, trauma, hypertension, diabetes, and Cushing's syndrome [8]. Among the 10 cases observed in the literature, 3 had hypertension, and incidental discovery were also common among them. Upon examination, the patient's body mass index in the current case was 31.1 kg/m^2^, which is an indication of obesity, and he also had hypertension. There are different theories about the cause of adrenal myelolipoma. Some authors suggest that it may arise from the transformation of adrenal capillaries, while others propose that it may result from emboli originating from bone marrow or from adrenal embryonic remnants of hematopoietic elements. There is also a report of myelolipoma expressing (3;21) (q25;p11) translocation, which indicates that it may have a neoplastic origin related to hematologic elements [11].

Adrenal myelolipoma typically affects individuals between the ages of 50 and 70 and is not known to have a predilection for any particular gender. However, there is a higher incidence of this condition in the right adrenal gland (59.2%) compared to the left (25.3%). It is typically observed as a unilateral tumor that doesn't exhibit any symptoms of endocrine disorder. However, in around 10% of cases, bilateral masses have been reported [12]. After reviewing the genuine literature on AMLs larger than 10 cm, it was found that the mean age of those affected was 48 years old, and the majority of cases were unilateral with a right-side predilection [2,[9], [10], [11],[13], [14], [15], [16], [17], [18],^19^] (Table 1). In the current case, a 63-year-old individual with a unilaterally left-sided 12 cm mass on the adrenal gland was diagnosed.

**Table 1:** Summary of laparoscopic excision of Giant AMLs >10 cm.

First author	Country	Patient Age (Y)	Gender	Size (cm)	Blood loss volume(mL)	Body mass index (kg/m^2^)	Location of mass	Chief Complaint	Chronic disease	Surgical approach	Difficulty of Dissection	Does it convert to open?	Operation time (h)	Compli- cation	Hospital stay in total(d)
Tinozzi et al. [2]	Italy	61	M	16	NR	30.24	Rt adrenal	Rt.hypochondrium pain	Hypertension	Transperitoneal Laparoscopy	No	No	2.66	No	5
Liu et al. [9]	China	26	F	13.5	NR	27.89	Lt adrenal	Secondary amenorrhea	Schizophrenia	Transperitoneal Laparoscopy	No	No	N/A	No	N/A
Ahmed et al. [10]	Iraq	43	M	13	200	23	Lt adrenal	nonradiating left loin pain	No	Transperitoneal Laparoscopy	No	No	4	No	3
Katsimantas et al. [11]	Greece	66	F	16.5	NR	NR	Rt adrenal	Asymptomatic	Hypertension and Hypothyroidism	Transperitoneal Laparoscopy	No	No	2.43	No	3
Chaudhary et al. [13]	India	55	M	15	50-60	NR	Lt adrenal	Lt. upper abdominal pain	No	Transperitoneal Laparoscopy	No	No	3.5	No	3
Kumar et al. [14]	India	25	F	15	NR	NR	Rt adrenal	Mild abdominal pain, micturition, and irregular menstrual cycle	No	Transperitoneal Laparoscopy	No	No	N/A	No	4
Gadelkareem et al. [15]	Egypt	45	M	16	180	34.64	Rt adrenal	Asymptomatic	No	Transperitoneal Laparoscopy	No	No	3.75	No	N/A
Yamamoto et al. [16]	Japan	69	F	14.3	NR	NR	Lt adrenal	Asymptomatic	No	Transperitoneal Laparoscopy	No	No	N/A	No	N/A
Zulia et al. [17]	USA	50	M	15	NR	NR	Rt adrenal	Rt.flank pain	Hypertension and Diabetes	Transperitoneal Laparoscopy	No	No	N/A	No	N/A
Pogula et al. [18]	India	46	M	13	100	35	Rt adrenal	Vague discomfort in the abdomen	No	Transperitoneal Laparoscopy	Yes	No	1.83	No	4

AMLs are classified into 3 different categories based on the size of the mass: masses smaller than 4 cm are termed small AMLs; when the mass reaches 10 cm, it is described as large AMLs; and masses greater than 10 cm are described as giant AMLs. The majority of cases are small AMLs, which account for about 82% of total cases [10]. Myelolipomas are typically not associated with any hormonal or clinical symptoms (asymptomatic). However, there have been cases where they have been associated with hormonal imbalances, such as congenital adrenal hyperplasia (CAH) and adrenocorticotropic hormone (ACTH) secretion. These cases are rare, though, and most patients do not receive an assessment for dysfunction of the endocrine system [20]. Pain or symptoms may appear if the tumor is larger than 10 cm and compresses surrounding structures, or if it ruptures and causes hemorrhaging. Although it is an extremely rare occurrence, it accounts for about less than 1% of cases [10,21].

Abdominal pain was a common symptom among the 10 cases observed in the literature. However, 3 out of 10 cases with different sizes of 16, 14.3, and 16.5 cm, respectively, were asymptomatic even with such a large size. The current case was also asymptomatic, and he did not experience any abdominal pain.

The diagnosis of AML can usually be easily identified through imaging. The typical features can be identified through CT and MRI scans. The tumor is usually round and has a pseudo-capsule. The AMLs have a cloudy pattern of fat, which can be identified without contrast uptake. The imaging technique can identify the poor vascularization of the tumor as well as the solid strands or islets of contrast-enhancing myeloid tissue. The proportion of these 2 components determines the attenuation, which can be identified through a CT scan. In most cases, AMLs show an attenuation between −50 and −20 HU. When using an MRI, hyperintense on both T1 and T2-weighted images indicates components of macroscopic fat, while hypointense on T1 and moderately hyperintense on T2-weighted images, indicates hematopoietic elements. Sometimes, the diagnosis of a tumor can be challenging. This is especially true when there is a nearly equal distribution of adipose and myeloid tissue, or if the tumor consists mostly of myeloid tissue. In these instances, differential diagnoses to separate it from other adrenal cortical adenomas should be considered along with blood workups [10].

In this case, on the contrast-CT scan, a retroperitoneal heterogenous mass was found on the left side of the adrenal gland. Additionally, laboratory blood test results showed normal levels of adrenal hormones.

Regarding the management of AMLs, the treatment of choice for small, nonfunctioning, asymptomatic AMLs is conservative and involves surveillance through annual imaging [10]. Adrenalectomy is only recommended if the lesion causes symptoms, is larger than 4-7 cm, poses a high risk of rupture and bleeding, or is suspected to be malignant based on imaging studies. Significant tumor growth observed on imaging is often considered another indication for surgical removal of AML. According to the RECIST (Response Evaluation Criteria in Solid Tumors) criteria for tumor growth, an increase of 20% in the size of the mass during the 6–12-month follow-up period is generally deemed as a significant increase [2,8,10].

Open adrenalectomy is commonly used as the treatment of choice for giant myelolipomas (AMLs >10 cm) as well as in emergency cases where hemorrhage or rupture has occurred. On the other hand, the minimally invasive approach is often preferred for small AMLs [2]. In the 10 cases that were reviewed in the literature, it was discovered that transperitoneal laparoscopy could safely be used to approach AMLs that had diameters greater than 10 cm. It was also found that laparoscopic surgery was feasible since none of the reviewed cases had to be converted to open surgery. Various studies have reported cases where laparoscopic surgery had to be converted to open surgery. For instance, a case series conducted by Alkhalifa et al. on 5 patients, including 4 with myelolipoma and 1 with angiomyolipoma, reported that 1 of the 4 myelolipoma patients had to be converted from laparoscopy to open surgery. Similarly, Gadelkareem et al. reported 1 case of AML that had to be converted from laparoscopy to open surgery [22,23]. Additionally, in almost all cases, the mass was successfully removed with ease. Furthermore, there were no complications reported as a result of using laparoscopic techniques in any of the reviewed cases.

As a minimally invasive approach, laparoscopy offers multiple benefits over traditional open surgeries. These advantages include reduced postoperative complications, improved cosmetic results, and a shorter recovery time. Laparoscopy also reduces the length of hospital stay and the incidence of surgical site infection due to the smaller incision. Additionally, there is less blood loss and fewer drainage times associated with this approach. Furthermore, a smaller incision in minimally invasive procedures can help alleviate postoperative pain as well as minimize scarring. Therefore, laparoscopic surgery is more effective than open procedures in terms of the rapid rehabilitation of patients after surgery [8].

## Conclusion

Laparoscopy can be a successful method for managing AML, even when they are large in size.

## Patient consent

Consent has been taken from the patients and the family of the patients.
